# Cinnamyl Isobutyrate Decreases Plasma Glucose Levels and Total Energy Intake from a Standardized Breakfast: A Randomized, Crossover Intervention

**DOI:** 10.1002/mnfr.201701038

**Published:** 2018-08-21

**Authors:** Christina M. Hochkogler, Julia K. Hoi, Barbara Lieder, Nicole Müller, Joachim Hans, Sabine Widder, Jakob P. Ley, Veronika Somoza

**Affiliations:** ^1^ Christian Doppler Laboratory for Bioactive Aroma Compounds Faculty of Chemistry University of Vienna Althanstraße 14 1090 Vienna Austria; ^2^ Department of Physiological Chemistry Faculty of Chemistry University of Vienna Althanstraße 14 1090 Vienna Austria; ^3^ Symrise AG Muehlenfeldstraße 1 37603 Holzminden Germany

**Keywords:** blood glucose, cinnamyl isobutyrate, energy intake, satiety, serotonin

## Abstract

**Scope:**

Cinnamon is associated with anti‐obesity effects, regulating food intake, improving plasma glucose levels and lipid profiles in vivo. In the present study, the impact of cinnamyl isobutyrate (CIB), one constituent of cinnamon, on ad libitum food intake from a standardized breakfast and outcome measures of hormonal regulation of appetite were investigated.

**Methods and results:**

In this randomized, short‐term crossover intervention study, a 75 g per 300 mL glucose solution solely (control) or supplemented with 0.45 mg CIB was administered to 26 healthy volunteers. Prior to and 2 h after receiving control or CIB treatment, subjective hunger perceptions were rated using a visual analog scale. Food intake from a standardized breakfast was assessed 2 h after treatments. Plasma peptide YY_3–36_, glucagon‐like‐peptide1, ghrelin, and serotonin as well as plasma glucose and insulin were measured in blood samples drawn at fasting and 15, 30, 60, 90, and 120 min after treatment. CIB administration decreased total energy intake and delta area under curve plasma glucose by 4.64 ± 3.51% and 49.3 ± 18.5% compared to control treatment, respectively.

**Conclusions:**

CIB, administered at a 0.45 mg bolus in 75 g glucose–water solution, decreased ad libitum energy intake from a standardized breakfast and postprandial plasma glucose levels.

## 1 Introduction

Combating the rising prevalence of overweight and obesity, associated with an increased risk of developing various comorbidities, has become a worldwide challenge.^1^, ^2^, ^3^ Therefore, medical treatment of obesity may become inevitable, taking into account, however, that combined treatment strategies are regarded more effective than drug application solely. Reducing food intake via modulation of anorexigenic and orexigenic signals as well as enhancing energy expenditure are strategies to combat overweight and maintain a healthy body weight.^4^ Hence, there is considerable interest in identifying anti‐obesity agents, focusing on bioactive dietary compounds that target food intake and promote feelings of satiety.

A variety of beneficial health effects have been attributed to naturally occurring aroma compounds in herbs and spices, such as cinnamon.^5^ Obtained from the bark of *Cinnamomum* species, it has been used for centuries for treating ailments such as respiratory and digestive disorders.^6^ More recently, cinnamon and its constituents have gained attention for potential application under diabetic and obesity conditions. Chronic and acute cinnamon supplementation have been reported to decrease fasting and postprandial plasma glucose levels and to delay gastric emptying in healthy and diabetic subjects.^7^, ^8^ Apart from reducing gastric emptying, increased insulin sensitivity has been proposed to explain the hypoglycemic effect of cinnamon.^9^, ^10^, ^11^ Regarding the active principles of cinnamon responsible for its hypoglycemic effects, plant secondary metabolites of phenolic compounds, including catechins, epicatechins, or proanthocyanidins, have been associated with beneficial effects in insulin signaling as well as antioxidative properties.^12^, ^13^, ^14^

Moreover, some studies hint at an impact of cinnamon and constituents on mechanisms regulating satiety in humans.^8^, ^15^, ^16^, ^17^ Despite evidence suggesting beneficial long‐term effects of cinnamon on maintenance of body weight in humans, to our knowledge, scientific data regarding its short‐term impact on food intake and satiety is scarce and inconsistent.^15^, ^16^ A search of compounds linked to antidiabetic and anti‐obesity properties of cinnamon led to the identification of cinnamaldehyde, constituting 80–90% of the essential oil of cinnamon bark. Cinnamaldehyde administration has been associated repeatedly with body weight and lipid‐modulating effects in animal models, including reduced cumulative food intake and gastric emptying rates,^18^, ^19^ as well as decreased weight gain, plasma triglycerides, and free fatty acid.^20^ In addition, ameliorated blood glucose levels and glucose tolerance in mice have been reported for cinnamaldehyde.^18^

In addition to reduced fasting‐induced hyperphagia and upregulated mRNA expression of hypothalamic neuropeptides associated with satiety after chronic cinnamaldehyde supplementation (10 mg kg^−1^ body weight) in mice,^21^ cinnamaldehyde was also found to modulate the secretion of satiety‐related gut hormones, including peptide YY (PYY) or serotonin.^22^, ^23^

Taken together, evidence suggests that consumption of cinnamon, cinnamaldehyde, and possibly other bioactive cinnamon constituents might be a promising approach in helping to maintain a healthy body weight by affecting food intake, blood glucose levels, and body composition through a satiety‐enhancing impact.^17^ However, consumption of cinnamon is self‐limiting due to strong and unique aroma values. Moreover, dietary intake of cinnamaldehyde and additional possibly bioactive constituents might be insufficient for antidiabetic and anti‐obesity effects.^24^, ^25^ Frequent consumption of cinnamon in larger amounts, especially *Cassia cinnamon*, might increase the risk of exceeding daily intake limit of coumarin (0.1 mg kg^−1 ^body weight per day), which has been linked to hepatotoxicity and carcinogenic effects.^26^ Consequently, selecting individual bioactive cinnamon compounds might be a safer application option. Cinnamaldehyde, although repeatedly demonstrated to affect glucose and lipid metabolism in animal models, might also be limited in its consumption due to characteristic cinnamon odor and spicy flavor characteristics.^24^ Thermosensitive transient receptor potential cation channel A1 (TRPA1) channels are activated by cinnamaldehyde, thereby evoking nociceptive responses and painful sensations.^27^ Cinnamaldehyde has also been reported to cause skin irritation and allergic contact dermatitis in humans.^28^, ^29^

To identify further potentially bioactive and less spicy cinnamon‐derived constituents, the aroma compound cinnamyl isobutyrate (CIB), naturally occurring in the essential oil of cinnamon bark, was chosen for the present study. Here, we propose a satiating effect of CIB. Despite structural similarities with cinnamaldehyde, CIB is mainly known for its moderate fruity and sweet flavor descriptors.^30^ It has been categorized as “Generally Recognized As Safe” (GRAS) by Flavor Extract Manufacturers Association (FEMA),^31^ and added to the EU list of flavoring substances (EFSA; Regulation EU 872/2012).

As our main hypotheses, we studied whether a bolus dose of 0.45 mg CIB has an impact on the ad libitum energy intake from a standardized breakfast (primary outcome measure), subjective hunger perceptions, and on postprandial blood glucose levels (secondary outcome measures) in moderately overweight volunteers following an oral glucose tolerance test (OGTT) with or without CIB supplementation. Additionally, ghrelin, glucagon‐like‐peptide1 (GLP‐1), PYY_3–36_, and serotonin concentrations were measured in the plasma as orexigenic and anorexigenic markers. The crossover study design in this short‐term intervention trial was conducted based on previous works which reported an impact of other aroma active food constituents on food intake and hormonal regulation of satiety.^32^, ^33^

## 2 Experimental Section

### 2.1 Study Population

This human intervention study was carried out with 26 metabolically healthy, moderately overweight (BMI between 25 and 32 kg m^−2^) male human subjects aged 21 to 43 years. Recruitment criteria for volunteers additionally required no tobacco consumption, alcohol, or drug abuse. Written consent was obtained from all participants after detailed instructions regarding the intervention. The Ethics Committee of the University of Vienna authorized the study design (registration no. 00163) which followed the guidelines of the Declaration of Helsinki. To determine eligibility in terms of study participation, subjects were medically screened beforehand. Determination of a hemogram as well as analysis of liver enzymes (aspartate aminotransferase, alanine aminotransferase alkaline phosphatase, and γ‐glutamyl transpeptidase), blood lipids (triglycerides, total, LDL, and HDL cholesterol), creatinine, glomerular filtration rate, thyroid‐stimulating hormone, and blood glucose levels (after 12 h fasting as well as 1 and 2 h after an OGTT) were performed by a medical laboratory (Blutlabor Dr. Greiner, 1220 Wien, Austria). A urine test was carried out to exclude glucosuria. Body weight and height were recorded using a digital scale (Seca Bella 840, Germany) to an accuracy of 100 g and a stadiometer with a precision of 0.1% (Seca 213, Germany), respectively.

### 2.2 Study Design

Following the medical screening, participants of this open, randomized, controlled, crossover study were randomly allocated and required to attend two separate study days after an overnight fast. At the first visit, the control group received a glucose solution (75 g glucose + 300 μl ethanol) devoid of additives, whereas the intervention group received a glucose solution with CIB supplementation (75 g glucose + 300 μl ethanol + 0.45 mg CIB). Study groups were switched at the second visit after 1 week to ensure that each subject participates once in the control and once in the intervention group. CIB was not sensorically detectable in the glucose solution at the applied dose of 0.45 mg. A visual analog scale (VAS) was applied to determine subjective perceptions of hunger, which describes the food intake depending on energy depletion and nutritional status in contrast to appetite, characterizing the desire to eat.^34^ It was completed by study volunteers before and 2 h after receiving the glucose solution. To assess their ad libitum energy intake, a standardized breakfast providing an average of 12.1 MJ with a total of 335 g carbohydrates, 126 g fats, and 80 g proteins was served 2 h after the OGTT. The meal comprised four rolls, four slices of dark bread, four slices of ham and cheese, 100 g honey and strawberry jam, 80 g butter, 180 g berry yogurt, 50 g coffee cream, 20 g sugar as well as 200 mL of coffee or tea and water as desired. The quantity consumed was determined by weighing the remaining food, in order to calculate the ad libitum energy and macronutrient intake using the German Food Code and nutrient database “Bundeslebens mittelschlüssel,” according to Hochkogler et al.^32^ Apart from assessing the potential impact on satiation, which determines the size of a meal, participants were asked to document their diet using provided forms by estimating their food intake on both study days for 24 h post‐intervention to evaluate an influence on satiety, describing the inter‐meal period following the end of an eating episode.^35^ Analysis of the diet records was achieved by means of the software program nut.s (dato Denkwerkzeuge, nut.s science, v1.29.34; Austria) as described by Hochkogler et al.^36^

### 2.3 Blood Sample Collection

Venous blood samples were taken at six time points over the course of 2 h using a venous catheter. The first blood sample was drawn at fasting, further drawings followed at 15, 30, 60, 90, and 120 min after consumption of the glucose solution. EDTA‐coated tubes (Sarstedt, Germany) were used for sample collection in preparation for ghrelin, GLP‐1, PYY_3–36_, and serotonin measurement, whereas heparin‐ or fluoride‐coated tubes (both Sarstedt, Germany) were used to determine plasma insulin and glucose concentrations, respectively. Plasma was obtained by centrifugation of blood collection tubes for 15 min at 1800 × *g* at 4 °C.

Determination of PYY_3–36_ concentration in the plasma required addition of the serine protease inhibitor AEBSF (4‐[2‐aminoethyl benzene] sulfonyl fluoride; Merck Millipore, Darmstadt, Germany) as well as a dipeptidyl peptidase protease inhibitor (DPP IV; Merck Millipore, Darmstadt, Germany) to the whole blood sample before centrifugation. For ghrelin analysis, AEBSF was added to the blood samples as well, which was promptly followed by centrifugation and plasma acidification with hydrochloric acid (0.05 m, final concentration). Aliquots of all samples were stored at −80 °C until further analysis.

### 2.4 Glucose, Insulin, PYY_3–36_, GLP‐1, Ghrelin, and Serotonin Assays

Determination of glucose concentrations in plasma was carried out by means of a colorimetric assay kit (Cayman Europe, Tallinn, Estonia) with an intra‐assay variation of 4.6–8.1% and an inter‐assay variation of 1.7–11.3%, whereas an enzyme‐linked immunosorbent assay (ELISA) kit (DRG Instruments GmbH IASON, Graz, Austria) with an intra‐assay variation of 1.8–2.6%, an inter‐assay variation of 2.9–6% was used to measure insulin concentrations in the plasma. ELISA assays, purchased from DLD Diagnostika (Hamburg, Germany) and Merck Millipore (Darmstadt, Germany), were also performed to assess plasma serotonin levels (intra‐assay variation: 4.7–6%; sensitivity: 5 ng mL^−1^) as well as total GLP‐1 (intra‐assay variation: 1–2%; inter‐assay variation: 10–12%), PYY_3–36_ (intra‐assay variation: 7–15%; inter‐assay variation: 6–11%), and ghrelin (intra‐assay variation: 1.11–1.91%; inter‐assay variation: 5.18–7.74%) concentrations. All assays were implemented according to specifications given by the manufacturer's protocols.

### 2.5 Statistical Analysis

All statistical analyses were carried out using SigmaPlot 13.0 (Systat Software GmbH). Normality of data was determined by Shapiro–Wilk test and if not indicated otherwise, normally distributed data are shown as mean ± standard error of mean. For determination of a sample size of 44, a power analysis was performed using G*Power 3, based on energy intake as main outcome measure from a pilot study with crossover design. This pilot test (*n* = 4) was conducted with male volunteers, who received an OGTT with or without 0.45 mg CIB supplementation on two separate study days. Taking into account the percentage change of energy intake (−12.3 ± 28.3%), an effect size of 0.44 was calculated (paired sample *t*‐test). From a total of 50 screened subjects, 34 were eligible to participate in the study. After further dropouts in the course of the study, 26 volunteers completed the intervention.

To assess statistical differences between control and CIB groups for mean delta values at t*_x_*–t_0_ for glucose and insulin, a two‐way repeated measures ANOVA for time and treatment followed by a Student–Newman–Keuls post hoc test was performed. Delta area under curve (^Δ^AUC) was determined for glucose, insulin as well as GLP‐1, PYY_3–36_, and ghrelin plasma concentrations over time. To test differences between treatment groups, a paired Student's *t*‐test (one or two tailed) was applied.

Moreover, a paired Student's *t*‐test (one tailed) was carried out to examine a statistical decrease in ad libitum energy intake, macronutrient intake, and hunger perceptions after CIB treatment compared to control treatment. For statistical analysis of energy and macronutrient intake assessed by food records, a paired Student's *t*‐test was performed as well.

## 3 Results

### 3.1 Total ad libitum Energy and Macronutrient Intake from Breakfast and Perceptions of Hunger

**Figure 1:**
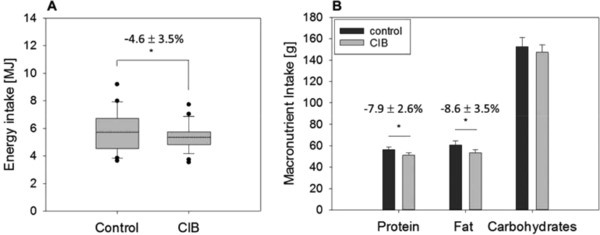
A) Total energy and B) macronutrient intake from a standardized breakfast 2 h after an OGTT administered solely (control) or with 0.45 mg cinnamyl isobutyrate (CIB) in 26 male healthy subjects. Statistical analysis (*p* ≤ 0.05) was conducted by a paired Student's *t*‐test (one tailed). Means are presented as dotted lines.

To determine the ad libitum energy intake, participants received a standardized breakfast 2 h after administration of a glucose solution with or without supplementation of 0.45 mg CIB. In the control group, a mean total energy intake of 5.81 ± 0.28 MJ was demonstrated compared to 5.37 ± 0.19 MJ in the intervention group, revealing a difference (−4.64 ± 3.51%; *p* = 0.03) in food intake (**Figure** 1A). Administration of CIB also showed a reduction in fat intake by 8.63 ± 3.53% as well as in protein intake by 7.91 ± 2.65% in comparison to the control treatment (Figure 1B). However, no changes in carbohydrate intake between treatments were detected.

**Figure 2:**
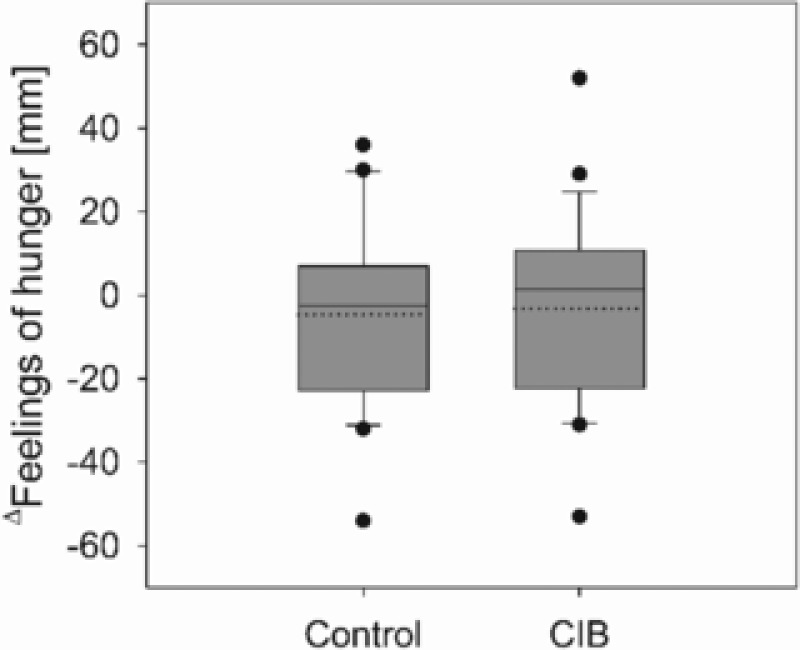
Mean Δ values of self‐reported hunger perceptions assessed by a 100 mm visual analog scale before and 2 h after an OGTT with or without (control) 0.45 mg cinnamyl isobutyrate (CIB) supplementation (*n* = 26). Statistical difference (*p* ≤ 0.05) was determined by a paired Student's *t*‐test (one tailed). Mean is presented as dotted line.

A continuous VAS, 100 mm in length, was used by participants to rate their subjective hunger perceptions before and 2 h after administering glucose solution with or without CIB supplementation. As presented in **Figure** 2, there was no significant difference in the feeling of hunger.

### 3.2 Energy and Macronutrient Intake from a 1 Day Food Record

Assessment of the dietary intake of study participants was achieved by keeping a 1 day estimated food record. CIB and control group did not differ in energy or macronutrient intake on day 1 (**Table** 1), apart from a decrease in saturated fatty acids (60.9 ± 4.27 control vs 53.5 ± 3.22 CIB, *p* = 0.05).

**Table 1:** Energy (MJ day^−1^), macronutrient (g day^−1^), and fatty acid (g day^−1^) intake after administration of 75 g glucose without (control) and with supplementation of 0.45 mg cinnamyl isobutyrate determined by an estimated food record over a period of 24 h post‐intervention. Values are presented as mean ± SEM. For statistical analysis, a one‐sample Student's *t*‐test (one tailed) was performed (*p* ≤ 0.05)

	Control	Cinnamyl isobutyrate	*p*
Energy	12.2 ± 0.65	11.9 ± 0.64	0.34
Protein	128 ± 10.6	121 ± 8.57	0.23
Carbohydrates	311 ± 23.3	294 ± 21.9	0.21
Fat	122 ± 7.81	111 ± 7.33	0.12
SFA	60.9 ± 4.27	53.5 ± 3.22	0.05
MUFA	38.9 ± 2.79	36.9 ± 2.99	0.27
PUFA	16.3 ± 1.68	15.2 ± 1.56	0.29

### 3.3 Plasma Concentrations of Glucose and Insulin

**Figure 3:**
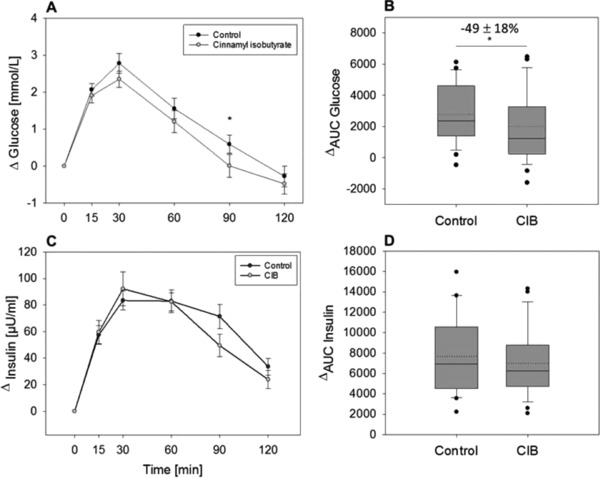
A) Mean Δ plasma glucose concentrations and B) AUC values as well as C) mean Δ plasma insulin concentration D) AUC values before and 15, 30, 60, 90, and 120 min after an OGTT with or without (control) 0.45 mg cinnamyl isobutyrate (CIB) supplementation (*n* = 25–26). Values are demonstrated as mean ± SEM. For statistical analysis, a two‐way repeated measures ANOVA for time and treatment (A,C) was performed. Significant differences (*p* < 0.05) between control and intervention treatments for each time point are marked with “*”. To assess the difference between AUC values (B,D), a paired Student's *t*‐test (one or two tailed) was performed (*p ≤ 0.05). Mean is presented as dotted line.

Ninety minutes after administering a 75 g glucose solution supplemented with CIB, reduced delta glucose levels of 0.01 ± 0.31 mmol L^−1^ were calculated compared to 0.59 ± 0.24 mmol L^−1^ in the control group (*p* = 0.02; **Figure** 3A). No difference between treatment groups (control vs CIB) could be shown at any other time point. Moreover, ^Δ^AUC values (2765 ± 371.3 control, 1988 ± 434.4 CIB, *p* = 0.03) demonstrated a decrease in plasma glucose concentrations after CIB intervention by 49.3 ± 22.8% compared to control treatment (Figure 3B). No significant differences, however, were shown for plasma insulin levels between treatment groups at any time point (AUC: 7663 ± 711.9 control vs 7000 ± 641.7; Figure 3C,D).

### 3.4 Plasma Ghrelin, PYY_3–36_, and GLP‐1 Levels

**Figure 4:**
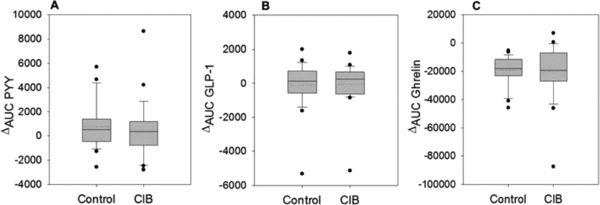
Mean ^Δ^AUC values for A) PYY_3–36_ , B) GLP‐1 and C) ghrelin concentrations from baseline levels 15, 30, 60, 90, and 120 min after administration of 75 g glucose without (control) and with supplementation of 0.45 mg cinnamyl isobutyrate (CIB) (*n* = 24–26). Values are shown as mean ± SEM. Statistical difference (*p* ≤ 0.05) was determined by a paired Students *t*‐test (one tailed). Mean is presented as a dotted line.

Ghrelin, PYY_3–36_, and GLP‐1 levels were assessed as short‐term markers of hunger and satiety before and at five time points after the OGTT (t_15_, t_30_, t_60_, t_90_, t_120_). As presented in **Figure** 4A,B, ^Δ^AUC values for PYY_3–36_ and GLP‐1 levels in the plasma did not differ between CIB and control treatment. Administration of CIB also did not affect plasma concentrations of ghrelin, as no difference could be demonstrated for ghrelin concentrations between the two treatment groups (Figure 4C).

### 3.5 Plasma Serotonin Concentrations

**Figure 5:**
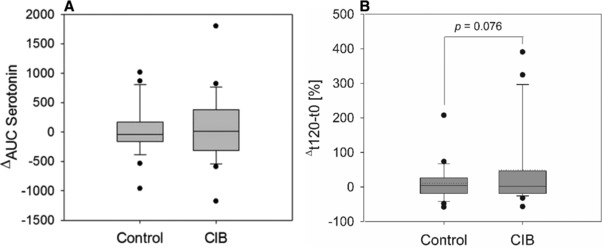
A) Mean ^Δ^AUC values for serotonin concentrations from baseline levels 15, 30, 60, 90, and 120 min after administration of 75 g glucose without (control) and with supplementation of 0.45 mg cinnamyl isobutyrate (CIB) and B) percentage changes of plasma serotonin levels from fasting to t_120_ (*n* = 26). Values are shown as mean ± SEM. Statistical difference (*p* ≤ 0.05) was determined by a paired Students *t*‐test (one tailed). Mean is presented as a dotted line.

Results of serotonin concentrations determined in the plasma after CIB treatment in comparison to control treatment are shown in **Figure** 5A. Statistical analysis revealed no difference in ^Δ^AUC values between treatment groups. Two hours after administration of CIB, calculated percentage changes of plasma serotonin levels from fasting to t_120_ showed a statistical trend (10.36 ± 10.06 control vs 49.36 ± 22.82 CIB; *p* = 0.07) to be higher than after receiving glucose solution solely (Figure 5B).

## 4 Discussion

In this crossover human intervention trial, a 0.45 mg bolus administration of CIB reduced ad libitum energy intake from a standardized breakfast and plasma glucose levels in moderately overweight men.

Cinnamon has previously been linked to beneficial health properties, including improved blood glucose levels,^13^, ^15^, ^37^, ^38^ insulin sensitivity^39^, ^40^ as well as serum lipid profiles,^7^ and to maintenance of body weight and body composition.^15^, ^16^ In terms of hypoglycemic effects of cinnamon, high concentrations of antioxidative phenolic compounds like proanthocyanidins might contribute to its beneficial effects on postprandial glucose levels.^13^, ^14^ Reports of other active ingredients, with the exception of cinnamaldehyde, or molecular pathways responsible for the above‐mentioned effects are relatively unknown and inconsistent. Anti‐obesity and antidiabetic properties of cinnamon are often attributed to its major flavoring component, cinnamaldehyde, which has been shown to reduce short‐term energy intake and cumulative body weight in animal models.^18^, ^23^ Besides a delay in gastric emptying rates, which has been associated with reduced hunger and increased satiety,^19^ regulation of satiety‐ and appetite‐related hormones might play a role in the cinnamaldehyde‐induced effect on energy intake.^18^, ^22^, ^23^ Overall, literature evidence suggests more pronounced effects after administration of individual constituents like cinnamaldehyde compared to cinnamon powder or extract. However, knowledge of anti‐obesity or antidiabetic activities of minor cinnamon‐derived compounds is sparse. In addition, usage of cinnamon and its major ingredient cinnamaldehyde is also restricted due to its strong spicy flavor and pungency, and potential hazardous impact from chronic consumption of higher amounts.^24^, ^26^ Thus, in the present study, CIB, exhibiting structural similarities to cinnamaldehyde, whereas presenting a less spicy, no pungent, and only a weak cinnamon‐specific taste and odor, was examined for its impact on short‐term energy intake. Here, bolus administration of CIB reduced short‐term energy intake from a standardized breakfast compared to control intervention by 4.64 ± 3.51%. As indicated by our results, this small, but significant decrease in energy intake can be attributed to a decline in fat and protein intake by 8.63 ± 3.53% and 7.91 ± 2.65%, respectively, whereas consumption of carbohydrates from the standardized meal was not affected. Despite administering CIB as a bolus in a low amount of 0.45 mg compared to effective short‐term applications of cinnamaldehyde in animal studies, ranging from 10–250 mg kg^−1^ body weight,^18^, ^23^ a reduction in food intake was seen in this study. However, the high number of dropouts leading to reduced sample size may have resulted in an inadequately powered study. This is a restricting factor for interpreting our outcomes which have to be verified in larger intervention studies to come. Whether the effect size might increase dose dependently as well, as it has been shown for cinnamaldehyde,^18^, ^22^ also needs to be investigated in future studies.

According to our main objective, we hypothesized that a decrease in short‐term energy intake is determined by decreased hunger perception. In contrast to this hypothesis, the slight reduction in food intake after CIB intervention was not accompanied by equally reduced hunger perceptions, as differences between treatment groups assessed by VAS were not detectable. It is not clear why CIB intake did not decrease hunger perceptions in the present study. Hlebowicz et al.^8^ demonstrated that adding 6 g cinnamon to a 300 g rice pudding delayed gastric emptying, suggesting extended post‐meal satiety due to increased gastric distension, but also did not affect satiety scores, estimated at several time points before and after the start of the meal. In addition, bolus administration of CIB did not affect cumulative food intake over a period of 24 h post‐intervention, as demonstrated by analyzing total energy intake by an estimated food record for 1 day.

Short‐term antidiabetic effects of cinnamon and its main constituent cinnamaldehyde have been demonstrated repeatedly,^8^, ^18^ suggesting increased glucose utilization and insulin activity in animal models after long‐term cinnamon administration.^39^ In this study, the impact of 0.45 mg CIB on plasma glucose concentrations was analyzed over a time course of 2 h after performing an OGTT with or without CIB supplementation. Administration of CIB reduced ^Δ^AUC levels compared to control treatment. Examination of the time course of glucose levels revealed a significant reduction 90 min after the OGTT compared to the control treatment, indicating an impact of CIB on glucose metabolism. In contrast to decreased glucose levels, insulin responses showed no changes at any time point between control and CIB treatment. These results are in accordance with another study demonstrating decreased postprandial glucose levels after bolus administration of 6 g cinnamon.^8^ Likewise, chronic oral administration of cinnamaldehyde was also reported to decrease blood glucose levels in diabetic rats (20 mg kg^−1^),^41^ and in obese mice (250 mg kg^−1^).^18^ In addition, Solomon et al.^40^ hypothesized delayed effects of cinnamon supplementation on insulin response and sensitivity, which was detected 2 weeks after daily cinnamon supplementation (6 × 500 mg), as opposed to a more immediate impact on postprandial glucose response. In contrast to high doses of cinnamon and cinnamaldehyde applied in previous studies, bolus administration of the comparatively small amount of 0.45 mg CIB significantly decreased postprandial plasma glucose levels in the present study. However, whether a long‐term administration enhances the demonstrated hypoglycemic effects or alters insulin responses need to be addressed in future studies.

In order to provide further insights into a potential appetite‐modulating impact of CIB, additional outcome measures associated with sensations of hunger and satiety were investigated. Short‐acting satiety signals in the gastrointestinal tract are generated primarily by gastric distension and the secretion of enteroendocrine peptides as mediators of intestinal satiety signaling.^42^ Anorexigenic peptides released in response to food intake include GLP‐1 and PYY, which are produced mainly by intestinal L‐cells.^42^ In addition, ghrelin levels, which are known for appetite‐inducing effects, were assessed in the present study. However, contrary to cinnamon and cinnamaldehyde, whose administration resulted in modulating effects on the secretion of anorexigenic hormones,^23^, ^38^ no differences in GLP‐1, PYY_3–36_, or total ghrelin levels were found in this study.

Having been shown to exert inhibitory effects on appetite, to generate feelings of satiety, and leading to suppression of food intake,^43^, ^44^ serotonin is also considered a marker of satiety. Although peripheral serotonin is not able to cross the blood–brain barrier, it has been shown repeatedly in vivo that peripheral administration also resulted in reduced food intake and accelerated satiety.^32^, ^45^, ^46^, ^47^ Whereas cinnamaldehyde has been shown to enhance serotonin release from enterochromaffin cells in vitro via TRPA1 stimulation,^22^ plasma levels of serotonin did not change after a CIB bolus in the present study. However, 2 h after receiving the glucose solution supplemented with CIB, a trend for a serotonin increase (10.3 ± 10.0% control vs 49.3 ± 22.8% CIB, *p* = 0.076) compared to control treatment was demonstrated. Future studies need to elucidate whether a more pronounced decrease in food intake at higher or frequent CIB supplementation in studies with larger sample sizes show a regulation on hormonal level. In addition, an involvement of TRPA1 has been discussed to be involved in cinnamaldehyde‐induced hormone release in the gastrointestinal tract.^22^, ^23^, ^48^ A TRPA1‐independent mode of action of cinnamaldehyde and potentially other cinnamon‐derived constituents concerning its impact on food intake cannot be excluded, but needs to be addressed in future studies as well.

Postprandial secretion of selected anorexigenic and orexigenic hormones as well as subjective hunger perceptions were not affected by CIB, although energy intake was reduced. Apart from energy expenditure, meal initiation and meal size not only depend on the nutritional status but also other signals, including palatability traits affecting taste and smell, influence our food intake.^35^, ^49^ Hunger perceptions, consequently, must be distinguished from appetite, which, by contrast, describes the desire to eat and is stimulated by availability of food and pleasure of eating.^34^ Thus, based on the results of the present study, we hypothesize an effect of CIB on energy intake and appetite, but cannot suggest a long‐term satiating impact of CIB, which is in accordance with other studies related to a satiating impact of cinnamon conducted in humans.^8^, ^50^

In conclusion, our study results point to a short‐term satiating effect of 0.45 mg CIB, corresponding to 1.5 ppm, an amount, which is used in nonalcoholic beverages, on energy intake. However, taking into consideration the small sample size as a limitation of this study, our results warrant larger intervention trials. These future studies will also need to elucidate dose‐dependent long‐term effects of CIB on energy intake and outcome measures of satiety to verify its potential activity as an anti‐obesity agent that might help to reduce food intake, and to maintain a healthy body weight and body composition.

## Conflict of interest

The authors declare no conflict of interest.
